# Predicting m-health acceptance from the perspective of unified theory of acceptance and use of technology

**DOI:** 10.1038/s41598-023-50436-2

**Published:** 2024-01-03

**Authors:** Marvello Yang, Abdullah Al Mamun, Jingzu Gao, Muhammad Khalilur Rahman, Anas A. Salameh, Syed Shah Alam

**Affiliations:** 1 Faculty of Entrepreneurship, Institute of Technology and Business Sabda Setia Pontianak, Kota Pontianak, Kalimantan Barat 78121 Indonesia; 2 UKM - Graduate School of Business, Universiti Kebangsaan, Malaysia, UKM, 43600 Bangi, Selangor Darul Ehsan Malaysia; 3 Faculty of Entrepreneurship and Business, Universiti Malaysia Kelantan, Pengkalan Chepa, Malaysia; 4 Angkasa-Umk Research Academy, Universiti Malaysia Kelantan, Pengkalan Chepa, Malaysia; 5 College of Business Administration, Prince Sattam Bin Abdulaziz University, 11942 Al-Kharj, Saudi Arabia

**Keywords:** Psychology, Human behaviour

## Abstract

Addressing the growing popularity of mobile health (m-Health) technology in the health industry, the current study examined consumers’ intention and behaviour related to the usage of digital applications based on the unified theory of acceptance and use of technology (UTAUT). In particular, this study quantitatively assessed the moderating role of perceived product value and mediating role of intention to use m-Health application among Indonesians. This study adopted a cross-sectional design and collected quantitative data from conveniently selected respondents through an online survey, which involved 2068 Telegram users in Indonesia. All data were subjected to the analysis of partial least square- structural equation modeling (PLS-SEM). The obtained results demonstrated the moderating effect of perceived product value on the relationship between intention to use m-Health application (m-health app) and actual usage of m-Health app and the mediating effects of intention to use m-Health app on the relationships of perceived critical mass, perceived usefulness, perceived convenience, perceived technology accuracy, and perceived privacy protection on actual usage of m-Health app. However, the intention to use m-Health app did not mediate the influence of health consciousness and health motivation on the actual usage of m-Health app. Overall, this study’s findings on the significance of intention to use m-Health app and perceived product value based on the UTAUT framework serve as insightful guideline to expand the usage of m-Health app among consumers.

## Introduction

Due to policies of social alienation norms and movement restrictions, digital technologies can facilitate strategies and responses to Covid-19 in remote ways that are difficult to achieve manually, which in turn leads to increased demand and use of digital technologies and applications such as online education and healthcare services, meaning that the Covid-19 pandemic leads to an inevitable surge in the use of digital technologies^1–3^. The adoption and expansion of digital applications have been significantly remarkable since the COVID-19 outbreak, and the most common are e-wallets, online study, and online healthcare-related applications^2,4,5^. There is a positive relationship between implementation of 4.0 digital application and the resilience of healthcare organizations to disruptive event through supply chains that contribute to patient diagnosis, patient treatment, and patient follow-up^6,7^. For instance, e-Doctor, which provides consultation services in healthcare management and biomedical research fields, has been integrated into the healthcare delivery system, particularly in developing countries^8^. M-Health app is a novel healthcare solution that is both cost- and time-effective^9^. Zhang et al.^10^ highlighted the relevance of developing m-Health app in educating consumers to make use of digital healthcare services. Exploring this phenomenon provides valuable insights on patients’ expectations and perceptions of using m-Health app.

Focusing on m-Health app, Marin et al.^11^ reported that m-Health app addresses the distance barriers between doctors and patients. Considering the significance of m-Health app, the government of Indonesia has actively supported digital behaviour and expanded the development of m-Health app. Through government support, consumers are more informed and willing to make use of m-Health app^12^. For easier access to m-Health app, the government of Indonesia successfully raised USD 145 million in 2020 for the development of m-Health app system, including Halodoc, Medisafe, and alliances apps^13^. The government has made efforts to enhance and ease the use of m-Health app for the public to obtain healthcare support, such as 24/7 doctor consultation, the purchase and delivery of medicine, and reliable customer support^14^.

Accordingly, technology acceptance model (TAM) has been widely in numerous studies on mobile-based technology, specifically to examine the intention to use technology within the context of information systems^15^. According to TAM, perceived ease of use (PEOU) and perceived usefulness (PU) are two key predictors of technology acceptance. Despite the extensive use of TAM across diverse contexts of industries^16–18^, the model is deemed inadequate when it comes to elucidating consumers’ decision to adopt m-Health app considering the potential influence of intention and other determinants^19^.

Meanwhile, the unified theory of acceptance and use of technology (UTAUT), a modified version of TAM, discusses the influence of attitude towards technology use on consumer behaviour^20^. The underlying theoretical basis of UTAUT in digital behaviour lies in consumers’ adoption or usage of m-Health app^21^. Alazzam and Addo^22^ highlighted the influence of behavioural intention on the adoption of technology behaviour, such as m-Health app. There have been limited studies on the factors that influence m-health adoption, such as the analysis of senior consumers in Bangladesh^20,23^ and the customer-producer relationships^24^. Alam et al.^25^ analyze price value and perceived reliability. Another study is limited to a review of the literature^26^ or to several nations^27^. Nonetheless, a study involving both perceived product value as mediating variable would be valuable for gaining a comprehensive understanding. The UTAUT has been widely utilized in research on technology adoption in health-related industries with respect to a number of factors, including cost and interaction aspects^28^, situational challenges and health concerns^29^ and customer attitude and condition^30^. However, these studies were not conducted specifically with m-health consumers perceived product value as moderation, intention as mediation and behavior were limited to Eastern countries. In light of the limitations of existing studies of m-health technology adoption, the researchers wish to examine the influence of certain the potential factors of adopting on behavioral intention in the context of Indonesia during the COVID-19, a potential and emergent nation with the fourth-largest population with respect to UTAUT approach. Consequently, the purpose of this study was to examine the applicability of the UTAUT model to the adoption of m-Health applications. In particular, we sought to address the following research questions:

- *RQ1* Which factors are crucial for the adoption of m-Health applications in Indonesia?
- *RQ2* Is the UTAUT model applicable for predicting users' intents to utilize an m-Health application?

In view of the above, it is undeniably important to explore consumer behaviour, specifically the adoption of mobile applications, from the perspective of UTAUT^12^. Addressing that, the current study used the UTAUT framework to assess the influence of health consciousness, health motivation, perceived critical mass, perceived usefulness, perceived convenience, perceived technology accuracy, and perceived privacy protection on the actual usage of m-Health app. Unlike prior studies, this study examined the moderating role of perceived product value and mediating role of intention to use m-Health app among consumers in Indonesia. This study was expected to present significant theoretical and practical insights on the significant factors that influence consumers’ intention to use m-Health app and actual usage of m-Health app. In the next section, the study will discuss the review of the literature and the theory underpinning the study and also the methods used in data analysis. The results and discussion will provide theoretical and practical implications of this study.

## Literature review

### Unified theory of acceptance and use of technology

The combination of theory of reasoned action (TRA), TAM, motivational model (MM), theory of planned behaviour (TPB), and social cognitive theory (SCT) has led to the development of UTAUT. Venkatesh et al.^31^ introduced UTAUT with four main factors that influence intention and usage of information technology namely performance expectancy, effort expectancy, social influence, facilitating conditions. The UTAUT as a holistic framework to adopt technology, which has been adopted in numerous recent studies on technology acceptance^20,32^. Moreover, Venkatesh et al.^20^ proposed UTAUT2 relating attitude by adding habit, hedonic motivation and price value as main constructs. However, this model received objection from Ramantoko et al.^33^ which exclude habit construct in the research model since mobile payment is a relatively new technology that has not yet gained sufficiently widespread use among consumers to generate a habit. Perceived price value in the research as less applicable in the m-payments context which usually causes no or very little additional financial cost to users (m-Health) and excluded^34,35^. Previous studies^36,37^ also exclude hedonic motivation since the researchers deemed the construct is not related to technology under investigation in context of m-health. In formulating the UTAUT 2 model, Venkatesh et al.^20^ argued that one would expect facilitating conditions to predict behavioural intention only if effort expectancy was not included in the model.

Among the studies that included moderators, few studies^20,38^ modeled the same four moderators as proposed by the original UTAUT model. There are two potential reason why prior studies may not have utilized moderators is because there may not be any variation in the moderator for the adoption and use context^38^. Second, the relationships proposed in the original UTAUT model may be reconsidered for completeness. Finally, the original UTAUT model may be reconsidered from the light of other constructs that may explain adoption and usage behaviors of individuals.

It is important to emphasize that since effort expectancy and performance expectancy in the UTAUT model are considered to represent the same constructs as perceived ease of use and perceived usefulness, many studies combined these similar constructs into one term^32,39^. This study attempts to develop the original of UTAUT in m-health app in context of Indonesian consumers. To avoid the double terms as previous studies done, this study also accords with earlier observations, which showed by Corduneanu^40^ pointed out that several intrinsic factors have been identified as important predictors of behavioral intention and adoption m-Health such as perceived technology accuracy, perceived privacy protection, and perceived product value in technology adoption models. This study adopted perceived usefulness, and perceive convenience as perceived ease of use and perceived usefulness^41^. Critical mass performs could be established when users believe that most of their colleagues are using the system which increases the likelihood of customer adoption^34^ and can be used to predict technology adoption^42^. Based on the previous studies definition of critical mass, the study adopted by Tan and Ooi^21^ critical mass performs as social influence which proposed by Venkatesh et al.^31^ or subjective norms proposed by Ajzen^43^. Facilitating conditions are defined as the degree to which an individual believes that an organizational and technical infrastructure exists to support use of the system^31^. Thus, perceived technology accuracy^25^ and privacy protection^44^ represent as technical infrastructures offered by the system to convince the users regarding accuracy and data safety as facilities of the m-Health app.

Hoque ^45^ demonstrates that perceived usefulness, perceived ease of use, subjective norms, and trust are critical to m-Health adoption to extended TPB. Alsyouf et al.^46^ found that perceived usefulness and perceived ease of use are the key factors influencing behavioural intention on m-Health app through TAM approach. Moreover, Cao et al.^47^ proposed perceived risk, trust, and health consciousness to extend the UTAUT on behaviour intention and found that trust is a significant role on behaviour intention to use m-Health. Facilitating conditions significantly affect technology adoption^48,49^. However, the results of Quaosar et al.^50^ indicated no significant effect of facilitation conditions on the intention to use health services. Thus, Mustaf et al.^32^ argued that it not only four constructs of UTAUT play significant attribute to intention and adoption on m-Health, however human attributes, economic factors, and social factors may impact how people perceive innovation technology. Previous studies^14,22,51^ in context to usage behavior often ignore the element of health consciousness attitude and health motivation in human nature. Shah et al.^52^ have found that the health consciousness and health motivation nature of humans are two significant factors behind behavioural intention. Furthermore, Rasul et al.^53^ found that health convenience is not significant factor behind human decision-making to adopt or reject new technology. This factor is neglected in the technology adoption models, and previous studies rarely investigate this factor concerning new technology adoption or rejection^20,54^.

Based on the inconsistent findings and theoretical gaps of previous studies on Table 1, the need for further study to gain better understand of user behavior and the factors influencing users’ intentions to use m-Health app in various context is required with the growing popularity of e-Health app, it is crucial to obtain good understanding on factors that can enhance the development and adoption of such applications^12^. This study proposes an integrated model of UTAUT examined health consciousness and health motivation on m-Health adoption, making it a perfect match for this study. Thus, this study is unique in that it extends the traditional UTAUT model framework by introducing health consciousness, and health motivation, perceived usefulness and perceive convenience, critical mass, technology accuracy and privacy protection, perceived product value, intention and adoption m-Health app. This model also highlights consumers’ acceptance of m-Health app, which has been extended to elucidate consumers’ acceptability and usage of technology. The current study focused on demonstrating how consumers perceive m-Health app and identify significant factors that can improve the actual usage of m-Health app in context of Indonesia.

**Table 1:** Key findings.

Author (s)	Research objectives	Literature approach	Methodology	Findings	Future research
Venkatesh et al.^31^	Proposed four main constructs of UTAUT model and four moderators	Review eight previous models to formulate Unified UTAUT	A questionnaire was created with items validated in prior research adapted to the technologies and organizations studied	UTAUT is a definitive model that synthesizes what is known and provides a foundation to guide future research in this area	1. Identify and test additional boundary conditions of the model 2. study the degree to which systems perceived as successful from an IT adoption perspective (i.e., those that are liked and highly used by users) are considered a success from an organizational perspective
Venkatesh et al.^20^	Proposed UTAUT2 (Hedonic motivation, price value, and habit)	A review of the extant literature by Venkatesh et al. (2003)	The target population was the current users of mobile Internet technology in Hong Kong with two methods to assess CMV that is Using PLS and CFA	Hedonic motivation, price value and habit play important role with three moderators (age, gender and experience)	Extend our model and examine potential interventions to foster or break habits in the context of continued IT use
Ramantoko et al.^33^ Tamilmani et al.^55^	Performance Expectancy, Effort Expectancy, Social Influence, Facilitating Condition, Hedonic Motivation, and Price Value Together but omitted habit	UTAUT 2 Approach	500 were distributed using face-to-face method, a more convenient method to address a larger sample population in a cost-effective manner and data was analyzed using Smart-PLS	Behavioral Intention toward adopting DHS could be described by factors Performance Expectancy, Effort Expectancy, Social Influence, Facilitating Condition, Hedonic Motivation, and Price Value significantly	This research did not explore the “intention to use” but merely on the intention to adopt
Koenig-Lewis et al.^35^	Perceived enjoyment, social influence, knowledge and perceived risk, and by identifying	The UTAUT 2 and Perceived price/value and habit were excluded as these are less applicable in the m-payments context	An online survey of 316 young people in France and the hypotheses were tested using structural equation modelling using AMOS 21	Perceived enjoyment, social influence, knowledge and perceived risk, play significant relationship after exclude price value and habit	The need to consider perceived risk of m-payments can be decreased by providing customers with an engaged and enjoyable experience
Mahfuz et al.^37^	UTAUT2 model with cultural dimensions and website quality to know the influences on the m-banking services adoption without hedonic motivation in the model	UTAUT 2	This paper analyzed by applied partial least squares (PLS) with 220 samples	Cultural dimension and web site quality to adoption of mobile banking in Bangladesh which is considered to be a vital consumer to adopt mobile banking services	Website quality and cultural dimension as a moderator variable like willingness to share on m-banking adoption
Dwivedi et al.^38^	Formalized an alternative theoretical model for explaining the acceptance and use of information system (IS) and information technology (IT) innovations	The acceptance and use of information system (IS) and information technology (IT) innovations	Combination of meta-analysis and structural equation modelling (MASEM) techniques. The meta-analysis was based on 1600 observations on 21 relationships coded from 162 prior studies on IS/ IT acceptance and use	The explanatory power of the theoretical model improved significantly when attitude is explicitly theorized	This research uncovered certain relationships that were not found in the original UTAUT model. Several of these paths identified in this research were due to the introduction of a new construct (attitude) not found in the original model
Hoque et al.^45^	Consumers’ health consciousness (HC), perceived knowledge (PK) and beliefs affecting the attitude and purchase intent (PI) of the consumers	Theory of Planned Behavior (TPB)	Survey conducted on a randomly selected sample of 712 households by using PLS-SEM	The results of the analyses corroborate that consumers’ health consciousness has a positive impact on perceived knowledge, belief, and attitude, but not on purchase intent	This study takes into account one dimension of health perception, namely physical health consciousness. Other dimensions such as mental, social, emotional, motivation and spiritual health have not been considered. The other important variables, such as the effect of emotion, specific belief, perceived risks, trust
Cao et al.^47^	UTAUT model to measure eight constructs: health consciousness, social influence, facilitation conditions, perceived risk, trust, performance expectancy, effort expectancy, and behavioral intention	UTAUT 2	A questionnaire survey in a Japanese university and collected 233 valuable responses and data Structural equation modelling was used for hypothesis testing	Four constructs of UTAUT have significant effect on behavioral intention. Health consciousness and social influence indirectly behavioral intention through trust	Future study needs to explore those who have prior experience with m-Health use and those who have no experience with mHealth use
Rasul et al.^53^	The UTAUT model explain the technology continuance of adopting m-Health	Technology acceptance model (TAM)	306 usable responses from a randomly crowdsource online survey of sugar-related mobile app users in Australia. Data were analyzed by structural equation modelling	Social influence was a significant predictor of continuance intention, but convenience was not, which could be attributed to increased app usage	The rich and refreshing insights revealed signal the potential fruitfulness of adding new factors, which future research can advocate or identify through critical
Alsyouf et al.^46^	Fills the literature gap regarding mediating effects of social influence and COVID-19 anxiety in the relationship between trust in government and exposure detection apps implementation, and between COVID-19 anxiety and exposure detection apps implementation, respectively	Technology acceptance model (TAM)	Quantitative study approach and a cross-section design targeted 586 participants from Saudi Arabia. The hypotheses were analyzed using the structural equation modelling–partial least squares (SEM-PLS3) approach	All constructs in this study found play significant impact on behavior except social media Awareness on app usage	Future research should consider different settings and include bigger samples representing a better result

### Development of hypotheses

According to the value-expectation theory, one’s evaluation of the outcomes of a particular behaviour and expectation of the behaviour influence one’s decision-making of the behaviour^56^. Dutta-Bergman^57^ highlighted the relationship between health consciousness and the selection of healthy lifestyle via psychological benefits and perceived naturalness. Meanwhile, Hoque et al.^45^ observed the positive perception of healthy lifestyle among health-conscious individuals. Health-conscious and educated consumers tend to examine food quality more carefully during their selection of foods^58^. In a more recent study, Iqbal et al.^59^ reported the positive influence of consumers’ attitude in terms of health consciousness and food safety concern on consumer engagement and the positive influence of consumer engagement on the intention to consume green product. Thus, the following hypothesis was proposed for testing in the current study:

#### H1

Health consciousness positively influences intention to use m-Health app.

Studies have discussed the influence of health motivation on health-related behaviour^60–62^. Extrinsic rewards, which come from family members, friends, or other social groups, potentially enhance consumer behaviour^63,64^. On the other hand, intrinsic rewards are associated with one’s personal satisfaction or positive inner feelings and experience towards a specific activity or behaviour^63^. Essentially, health motivation reflects the causes and processes of behaviour based on external information sources and internal cognitive features^62^. Consumers motivation effect user intentions to continue using m-Health app^65^. Thus, the following hypothesis was proposed for testing in this study:

#### H2

Health motivation positively influences intention to use m-Health app.

Lankton et al.^66^ described perceived accuracy as one’s belief that the technology functions as expected. Meanwhile, Oechslein et al.^67^ highlighted the influence of the use of news aggregators and ease of use on perceived accuracy, trusting beliefs, and perceived utility. The study further highlighted the positive influence of assessed accuracy on perceived usefulness and usage intention. According to Alam et al.^25^ perceived accuracy is crucial in the evaluation of competency and objectivity, communication of standards, relevance and appropriate use of technology, use of accurate data, and the presence of procedural and distributive fairness. In a more recent study, Ullah et al.^49^ highlighted the importance of management and employee perspectives on perceived accuracy as well as fear of technology, the necessity for employee participation with e-performance appraisals, and the requirement for clear communication of standards in a different study. In view of the above, the following hypothesis was proposed for testing:

#### H3

Perceived technology accuracy positively influences intention to adopt m-Health app.

Critical mass can be achieved when consumers observe that most of their peers have utilised the particular technology—this subsequently increases the possibility of actual adoption of the technology^18^. There are various factors that influence the relationship between beliefs and IT adoption, depending on the technology under consideration^21^. Additionally, critical mass is imperative for the sustainability of the technology system and serves as an indicator to stimulate collective actions towards new technology adoption^67^. Thus, the following hypothesis was proposed for testing in this study:

#### H4

Perceived critical mass positively influences intention to adopt m-Health app.

The transition from a conventional strategy to an online-based technological strategy has substantially changed the overall design and structure of organisational activities and employees’ perspectives of the sustainability of their company^49^. Hoffmann et al.^68^ found that lower level of concern about online privacy among Internet users who believe in the success of regulation enforcement. Addressing online privacy concerns, the implementation of more regulations maintains the impact magnitude of regulation^69^. Škrinjarić et al.^70^ discussed the internal and external factors that influence the intention to adopt online privacy protection technology among Internet users. Based on the review of these findings, the following hypothesis was proposed for testing:

#### H5

Perceived privacy protection positively influences intention to adopt m-Health app.

Wilson et al.^71^ described perceived usefulness as the general judgment of the time and effort required to learn and use a new product. Nayanajith et al.^72^ viewed perceived usefulness as the extent of one’s thoughts of online retail businesses increasing the value and efficacy of the products. In a more recent study, Lu et al.^73^ highlighted the potential influence of perceived usefulness on students’ intention to continue using the learning system and potential users’ feelings and decisions of adopting technological improvements. Rawashdeh et al.^74^ reported the significant perceived usefulness and usability on continuous use intention. Thus, the current study postulated the following hypothesis:

#### H6

Perceived usefulness positively influences intention to adopt m-Health app.

One of the notable features that mobile payments offer is convenience for consumers to acquire on-demand utility and make purchases effortlessly^73^ The study highlighted the significant role of perceived convenience in influencing consumers’ decision to use a new application. Pal et al.^75^ noted convenience as a single concept with multiple benefits. Mobile payment technology offers convenience that eases consumers in their daily tasks^74^. Such convenience exhibits considerable influence that can motivate the actual usage of the technology. Thus, the following hypothesis was proposed for testing:

#### H7

Perceived convenience positively influences intention to adopt m-Health app.

Shaw and Sergueeva^76^ highlighted the substantial influence of intention to use, among various predictors, on consumer behaviour. Qing and Haiying^77^ similarly supported the strong relationship between intention to use and actual adoption of new applications. Consumers who understand the value of such services, such as time-savings, transaction speed, and various purchasing possibilities, are likely to develop continuous usage intention^78^. Therefore, the current study proposed the following hypothesis:

#### H8

Intention to use m-Health app positively influences the actual usage of m-Health app.

### Moderating role of perceived product value

Perceived value is a widely known term that is associated with consumers’ purchase behaviour, and businesses make use of perceived value to gain higher competitive advantage^79^. Higher perceived value increases consumer satisfaction, consumer loyalty, and consumer retention, which ultimately leads to business success. The actual value of the price of a product reflects consumers’ desire for the product given its influence on consumers’ decision to purchase. Consumers are generally willing to purchase and pay more for beneficial products, such as green products^80^. Perceived value in terms of quality and pricing play an important role in ensuring high value for consumers^81^. Chinomona et al.^79^ empirically examined the influence of product quality on consumer behaviour on using the product examined the influence of product quality on consumer behaviour on using the product. Kim et al.^81^ identified consumers’ perceived value as a key criterion of consumer behaviour of using m-Health app based on the exchange hypothesis in marketing research. On a similar note, Kaczorowska et al.^80^ recognised product value as an essential indicator of consumers’ brand expectations and their decision of using the product. In a more recent study, Wang et al.^82^ demonstrated the significant influence of perceived value on consumers’ purchase intention. Based on the review of these findings, the following hypothesis was tested in this study:

#### H9

Perceived product value moderates the influence of intention to use m-Health app on the actual usage of m-Health app.

### Mediating role of intention to use m-health app

For actual adoption behavior, behavioral intention is one of the primary and most important predictors. Alam et al.^25^ highlighted the mediating role of intention in the relationship between various constructs and technology adoption and confirmed that higher health awareness and motivation lead to a tendency to use e-healthcare technologies that provide e-health services and health management to maintain personal health. Dehghani et al.^83^ confirmed that health awareness and motivation are specific preferences that motivate health behaviors through intentions. In studies employing TAM and UTAUT, numerous scholars have discussed the potential impact of other factors on the acceptance of new technologies and have found that technology-level attributes such as compatibility, privacy protection, usefulness, and accuracy remain meaningful predictors of intention to use and use the technology^44,51^. Considering that, the intention to use m-Health app potentially mediate the influence of health consciousness, health motivation, perceived critical mass, perceived usefulness, perceived convenience, perceived technology accuracy, and perceived privacy protection on the actual usage of m-Health app. In view of the above, the current study proposed the following:

#### H10

Intention to use m-Health app mediates the relationships of health consciousness, health motivation, perceived critical mass, perceived usefulness, perceived convenience, perceived technology accuracy, and perceived privacy protection with the actual usage of m-Health app.

**Figure 1:**
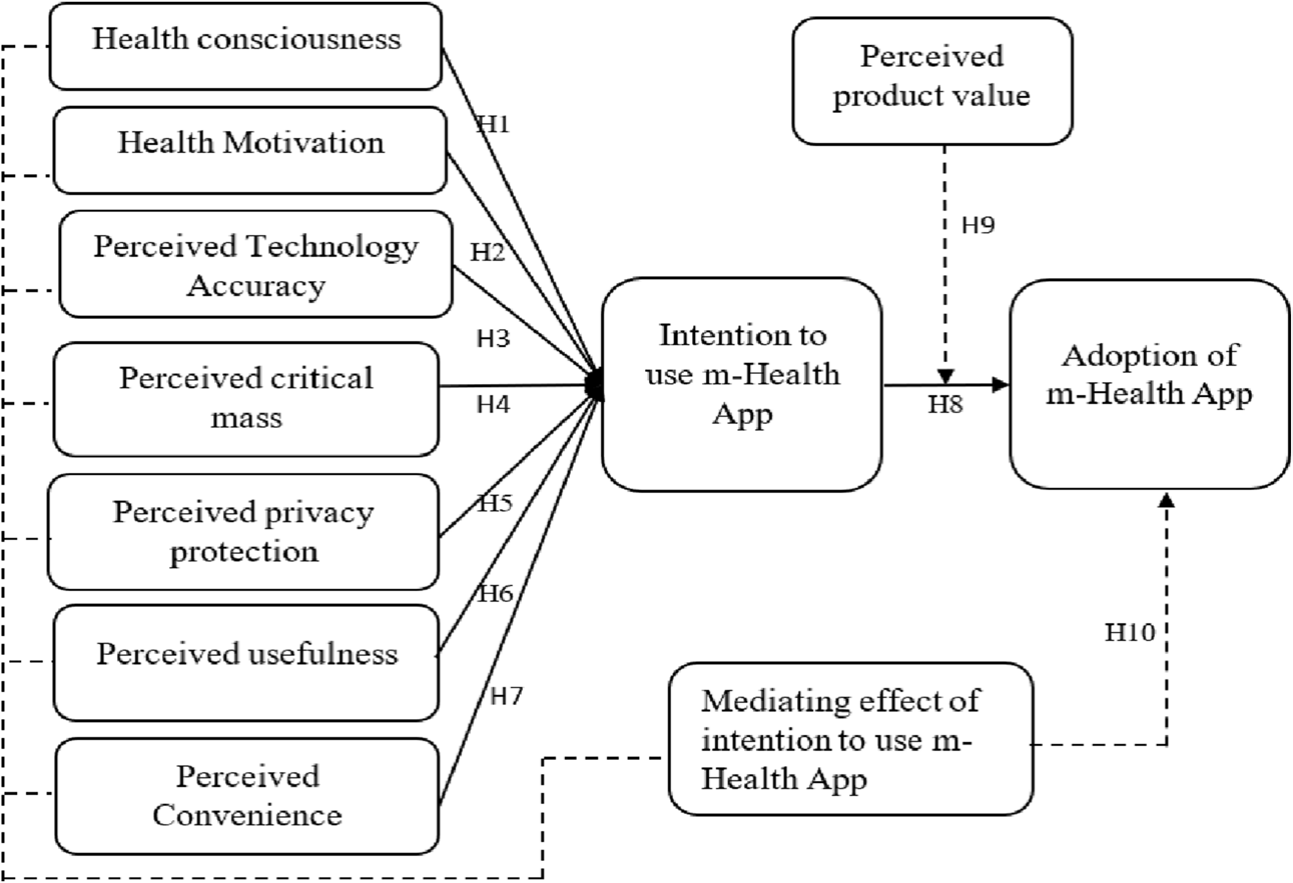
Conceptual model.

Based on the review of related literature, the current study’s conceptual model on the adoption of m-Health app is presented in Fig. 1.

## Methodology

### Data collection

This study targeted consumers with the experience of using m-Health app in order to obtain insights and feedback that can enhance the overall user experience of the app. The research ethics committee of Institute of Technology and Business Sabda Setia Pontianak, Indonesia approved this study (Approval No. 392A/ITBSS/V/2023). This study has been performed in accordance with the Declaration of Helsinki.

This study utilised Google Forms for the development of instrument and the collection of the online survey data. With more than 700 million users in 2022, Telegram is the most popular social media platform in Indonesia^84^. A Telegram group consists of users who can freely interact with one another and discuss similar topics of interest, and a user can simultaneously join multiple Telegram groups. This study uses G-power software was introduced by Faul et al.^85^ to determine the minimum sample size because it is unknown how many users employ m-Health app precisely. G-power statistical analysis software is used as a tool to calculate the minimum sample size of the research in order to determine the minimum sample size. According to the G-power statistic (effect size = 0.15; *α* = 0.05; *β* error prob = 0.95), the minimum sample size for this study is 153 samples. Therefore, an online survey link was shared to a total of 3,120 respondents across Telegram groups. Written informed consent for participation was obtained from respondents who participated in the survey (by clicking ‘I agree" to the informed consent statement in the form). The overall data collection process was conducted from 1 January 2022 to 15 July 2022. At the end of the data collection, this study successfully obtained 2,068 completed questionnaire sets.

Given that the objective of this study is to derive theoretical generalizability, not population generalizability, convenience sampling was employed. A smaller number (around 30 per cent) of via email surveys were distributed with convenience sampling at the same period of time to minimize potential sampling bias^86^. The questionnaire was then pre-tested using a convenience sample of 30 respondents (From March 2022 to June 2022). An independent sample t-test indicates that no significant differences can be found between the data that collected from the two sources. After completion, suggestions and comments were collected from respondents to identify potential errors in terms of the wording, phrasing and sequencing of questions, which were then corrected. Items with similar meaning, which could not be clearly distinguished, were eliminated. The 30 respondents in the pilot test were then excluded from the final data set.

### Constructs and measures

Health consciousness as one’s willingness to take responsibility and perform activities that benefit health or one’s motivation to engage in practices that promote good health^57^. As for the current study, five items from Dutta-Bergman^57^ adopted to measure health consciousness. Meanwhile, health motivation as one’s personal motivation to achieve health-related goals towards better health^63^. For the measurement of health motivation in this study, five items were adopted from Guo et al.^63^. Perceived critical mass as the minimum number of adopters to sustain the adoption rate of an innovation^21^. Five items were adopted from this prior study to measure perceived critical mass in this study. As for the measurement of perceived usefulness, five items were adapted from the study conducted by Tan and Ooi^21^. In addition, five items were adopted by Tan and Ooi^21^ to measure perceived convenience in the current study. The prior study defined perceived convenience as intrinsic motivation and expectation of using m-Health app. Perceived technology accuracy in this study referred to the confidence of performing tasks on a new innovation accurately and repeatedly. For this, five items were adopted from Alam et al.^25^. Likewise, five items were adopted from Gao et al.^44^ to measure perceived privacy protection. Perceived privacy protection involves identifying and classifying information that needs to be secured and protected. Adding to that, the current study adopted four items from Kim et al.^81^ to measure perceived product value. This previous study defined perceived product value as the expenses of effort and time and the consideration of both benefits and costs. The current study operationalised intention to use m-Health app as the extent of one’s willingness to use m-Health app. Five items were adopted from Alam et al.^25^ measure this construct. The single item of the usage of e-doctor apps in this study was adopted from Yang et al.^5^ and Hayat et al.^87^.

### Common method bias

The obtained results of the current study revealed that a single component accounted for 40.689% of the total variance, which did not exceed the recommended threshold of 50%^88^. According to Kock^89^ a model is not affected by common method bias if all variance inflation factors (VIFs) in full collinearity test are equivalent to or lower than 5. The values of VIF for health consciousness (1.970), health motivation (1.506), perceived technology accuracy (2.013), perceived critical mass (2.422), perceived privacy protection (1.301), perceived usefulness (2.358), perceived convenience (2.228), intention to use m-Health app (2.140), perceived product value (3.037), and actual usage of m-Health app (1.188) did not exceed the minimum threshold value of 3.3. Thus, the model in this study was free of common method bias.

### Multivariate normality

The online Web Power tool (source: https://webpower.psychstat.org/wiki/tools/index) was utilised in this study to examine the multivariate normality of the obtained data. The recorded Mardia’s *p*-values did not exceed 0.05, suggesting the potential issues of multivariate normality^90^. Therefore, PLS-SEM was deemed fitting for the current study to analyse the data ^91^.

### Data analysis

Using SmartPLS 4.0, PLS-SEM was performed. In particular, path models with latent constructs were evaluated^92^. This analysis is appropriate for the analysis of small datasets and composite models with no goodness-of-fit assessment^91^. The analysis typically involves two main steps, which were performed in this study. Firstly, the measurement model was assessed in terms of reliability and validity^92^. Following that, the structural model involved the use of bootstrapping procedure to test the proposed hypotheses^92^. The model accuracy was evaluated based on *r*^*2*^ and effect size of *f*^2^ (representing effect size).

## Results

### Demographic characteristics of respondents

Table 2 presents the demographic profile of respondents. In particular, the majority of respondents were women (54.9%) and of the age between 18 and 25 years (58.5%). The other respondents were of between 26 and 35 years of age (12.8%), followed by those in the age groups of between 36 and 45 years (10.6%), between 46 and 55 years (8.7%), between 56 and 65 years (8.4%), and finally, above 65 years (1.1%). As for the education level, most of the respondents were high school graduates (33.7%), followed by Bachelor’s degree holders (23.8%), Master’s degree holders (16.5%), doctoral degree holders (13.8%), and lastly, higher secondary school graduates (12.1%). Besides that, about 31.4% of the total respondents reported earning under Rp. 2.5 million per month, followed by Rp. 15,000,001 to Rp. 20,000,000 (20.9%), Rp. 2,500,001 to Rp. 5,000,000 (17.5%), Rp. 5,000,001 to Rp. 10,000,000 (11.4%), and Rp. 10,000,001 to Rp. 15,000,000 (10.1%). Only 8.7% of the total respondents reported earnings above Rp. 20,000,000. Last but not least, about 45.5% of the total respondents were residents of Java, followed by Kalimantan (16.5%), Sulawesi (14.4%), Papua (13.1%), and Sumatera (10.5%).

**Table 2:** Demographic respondents.

Gender	n	%	Monthly income	n	%
Male	933	45.1	< Rp. 2.5 million	649	31.4
Female	1135	54.9	Rp. 2,501,000–5,000,000	362	17.5
Total	2068	100	Rp. 5,000,001–10,000,000	236	11.4
			Rp. 10,000,001–Rp. 15,000,000	209	10.1
Age	*n*	*%*	Rp. 15,000,001–20,000,000	433	20.9
18–25 years	1209	58.5	Above Rp. 20,000,000	179	8.7
26–35 years	265	12.8	Total	2068	100
36–45 years	220	10.6			
46–55 years	179	8.7	*Province*	*n*	*%*
56–65 years	173	8.4	Java	941	45.5
> 65 years	22	1.1	Kalimantan	341	16.5
Total	2068	100	Sulawesi	298	14.4
			Sumatera	218	10.5
Education	n	%	Papua	270	13.1
Secondary school	251	12.1	Total	2068	100
High school	697	33.7			
Bachelor degree	493	23.8			
Master degree	342	16.5			
Doctoral degree	285	13.8			
Total	2068	100			

### Validity and reliability

Based on the recommendations of Hair et al.^91^ the current study evaluated the construct reliability of latent constructs based on Cronbach’s alpha coefficients, Dijkstra-Henseler’s rho, and composite reliability. The obtained results revealed that all values of Cronbach’s alpha exceeded 0.65^92^, with its lowest value of 0.734. The results further revealed that all values of Dijkstra-Henseler’s rho exceeded 0.70, with its lowest value of 0.74. Besides that, the results demonstrated that the values for composite reliability exceeded the minimum threshold value of 0.70^92^, with its lowest value of 0.833. All results are summarised in Table 3, which confirmed the reliability of the latent constructs.

**Table 3:** Validity and reliability.

Variables	No. items	Mean	Standard deviation	Cronbach’s Alpha	Dijkstra-Hensele's *rho*	Composite reliability	Average variance extracted	Variance inflation factors
HCS	4	4.44	0.504	0.734	0.765	0.833	0.559	1.672
HMO	4	4.39	0.568	0.753	0.762	0.843	0.573	1.496
PTA	4	4.28	0.497	0.752	0.784	0.841	0.573	1.298
PCM	4	4.22	0.628	0.781	0.784	0.860	0.606	1.567
PPP	4	3.05	1.088	0.893	0.985	0.922	0.748	1.117
PUS	4	4.32	0.563	0.805	0.808	0.873	0.631	1.635
PCN	4	4.27	0.537	0.806	0.814	0.873	0.632	1.702
UIA	4	4.21	0.536	0.737	0.740	0.835	0.558	1.377
PPV	4	4.26	0.566	0.792	0.794	0.865	0.615	1.691
ADA	1	4.04	0.820	0.900	0.876	0.970	0.806	

HCS: Health consciousness; HMO: Health Motivation; PTA: Perceived Technology Accuracy; PCM: Perceived critical mass; PPP: Perceived privacy protection; PUS: Perceived usefulness; PCN: Perceived Convenience; UIA: Use Intention of App; PPV: Perceived product value; ADA: Adoption of Use App.

**Figure 2:**
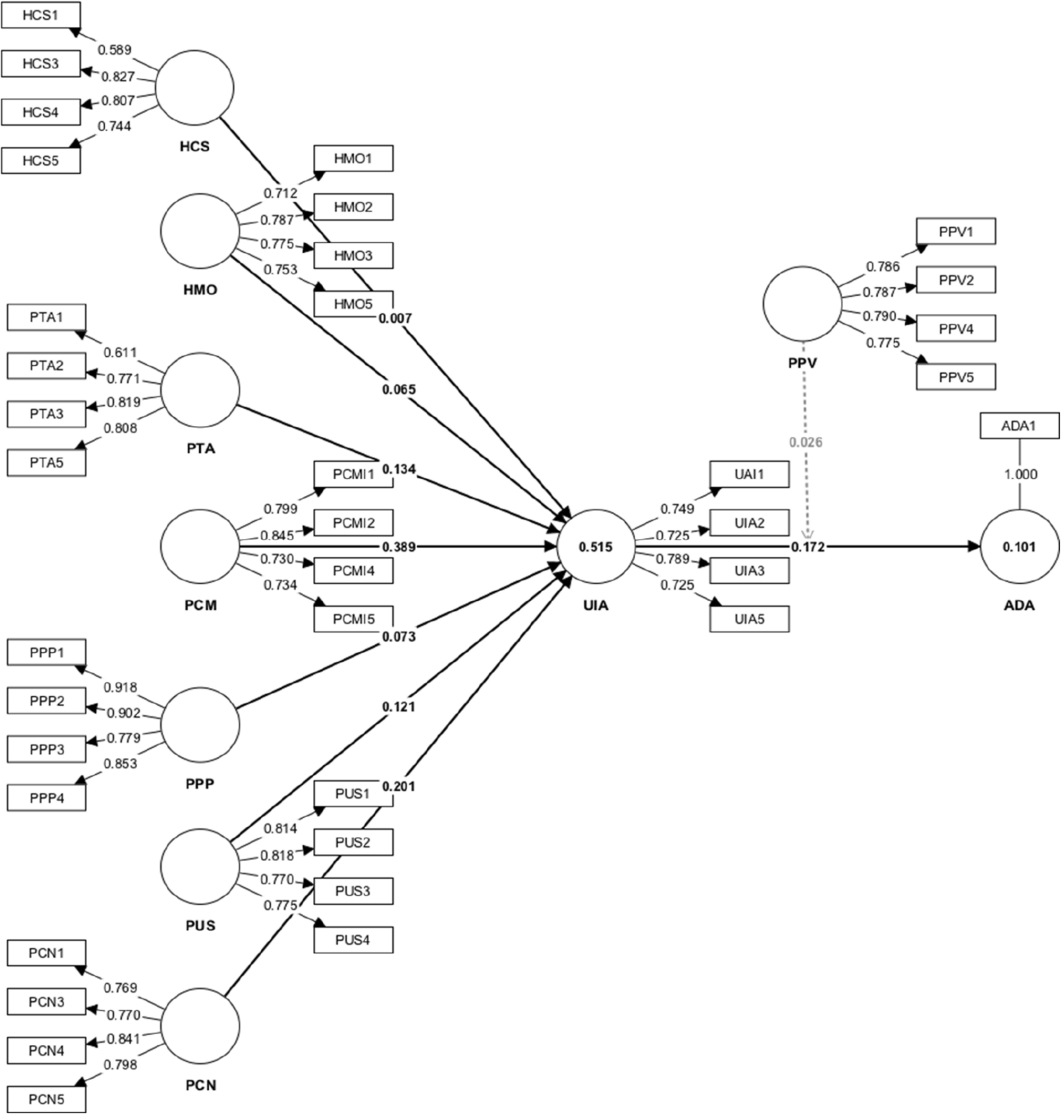
Measurement model.

Meanwhile, the convergent validity of the constructs was determined based on the average variance extracted (AVE). The obtained results demonstrated that all values of AVE exceeded 0.50^93^. In other words, each construct in this study exhibited adequate convergent validity, reflecting its unidimensionality. Adding to that, the values of VIF did not exceed the threshold value of 3.3, suggesting the absence of multicollinearity issues^92^. Figure 2 presents the measurement model of the current study.

Apart from convergent validity, the discriminant validity of the constructs was examined, specifically on the distinctiveness of latent variables. With that, this study examined the results of Fornell-Larcker criterion, Heterotrait-monotrait ratio, and loading and cross-loadings, which are presented in Table 4. Overall, the constructs demonstrated reliable loadings, reflecting appropriate discriminant validity for PLS-SEM.

**Table 4:** Discriminant validity.

Code	HCS	HMO	PTA	PCM	PPP	PUS	PCN	UIA	PPV	ADA
HSC1	*0.589*	0.306	0.039	0.156	0.476	0.124	0.022	0.213	0.184	0.193
HSC3	*0.827*	0.426	0.208	0.514	− 0.049	0.341	0.293	0.366	0.402	0.150
HSC4	*0.807*	0.477	0.219	0.487	− 0.130	0.415	0.377	0.335	0.430	0.150
HSC5	*0.744*	0.401	0.150	0.432	0.022	0.353	0.253	0.297	0.442	0.152
HMO1	0.283	*0.712*	0.214	0.193	− 0.056	0.246	0.118	0.225	0.153	0.062
HMO2	0.467	*0.787*	0.212	0.379	0.081	0.296	0.194	0.332	0.248	0.146
HMO3	0.364	*0.775*	0.168	0.371	− 0.104	0.263	0.284	0.304	0.246	0.067
HMO5	0.502	*0.753*	0.277	0.396	− 0.110	0.375	0.322	0.302	0.378	0.105
PTA1	0.118	0.112	*0.611*	0.159	0.095	0.290	0.477	0.270	0.371	0.167
PTA2	0.121	0.148	*0.771*	0.291	− 0.343	0.485	0.497	0.313	0.527	0.111
PTA3	0.286	0.303	*0.819*	0.433	− 0.208	0.545	0.428	0.442	0.484	0.159
PTA5	0.119	0.253	*0.808*	0.387	− 0.272	0.427	0.520	0.453	0.382	0.096
PCM1	0.381	0.391	0.364	*0.799*	− 0.172	0.478	0.397	0.547	0.456	0.138
PCM2	0.458	0.336	0.307	*0.845*	− 0.204	0.465	0.360	0.479	0.537	0.160
PCM4	0.435	0.358	0.309	*0.730*	− 0.183	0.425	0.342	0.466	0.397	0.124
PCM5	0.469	0.323	0.390	*0.734*	− 0.235	0.469	0.505	0.487	0.570	0.194
PPP1	0.036	− 0.080	− 0.215	− 0.252	*0.918*	− 0.284	− 0.224	− 0.107	− 0.169	0.150
PPP2	0.044	− 0.044	− 0.235	− 0.240	*0.902*	− 0.212	− 0.147	− 0.159	− 0.136	0.102
PPP3	0.120	0.016	− 0.218	− 0.101	*0.779*	− 0.195	− 0.093	− 0.053	− 0.063	0.135
PPP4	0.014	− 0.063	− 0.244	− 0.233	*0.853*	− 0.262	− 0.167	− 0.102	− 0.160	0.129
PUS1	0.291	0.321	0.505	0.434	− 0.209	*0.814*	0.382	0.442	0.457	0.266
PUS2	0.396	0.295	0.446	0.479	− 0.188	*0.818*	0.416	0.478	0.561	0.257
PUS3	0.370	0.353	0.445	0.556	− 0.304	*0.770*	0.508	0.423	0.548	0.184
PUS4	0.303	0.281	0.468	0.414	− 0.172	*0.775*	0.541	0.416	0.513	0.183
PCN1	0.254	0.244	0.657	0.461	− 0.250	0.518	*0.769*	0.508	0.573	0.168
PCN1	0.287	0.190	0.421	0.402	− 0.075	0.314	*0.770*	0.351	0.524	0.184
PCN1	0.282	0.282	0.426	0.410	− 0.169	0.494	*0.841*	0.462	0.552	0.163
PCN1	0.266	0.259	0.449	0.361	− 0.071	0.470	*0.798*	0.446	0.479	0.191
UIA1	0.315	0.303	0.398	0.370	0.013	0.337	0.299	*0.749*	0.450	0.196
UIA2	0.255	0.301	0.482	0.380	− 0.302	0.425	0.392	*0.725*	0.421	0.089
UIA3	0.284	0.292	0.568	0.474	− 0.114	0.408	0.514	*0.789*	0.517	0.224
UIA5	0.377	0.273	0.375	0.279	− 0.014	0.480	0.463	*0.725*	0.537	0.294
PPV1	0.389	0.291	0.464	0.495	− 0.059	0.469	0.639	0.550	*0.786*	0.254
PPV2	0.363	0.235	0.507	0.533	− 0.351	0.559	0.689	0.518	*0.787*	0.212
PPV4	0.375	0.283	0.489	0.516	− 0.233	0.595	0.485	0.514	*0.790*	0.213
PPV5	0.441	0.271	0.453	0.438	0.095	0.446	0.301	0.450	*0.775*	0.241
ADA1	0.208	0.129	0.171	0.198	0.142	0.282	0.221	0.274	0.295	*1.000*
Fornell-Larcker criterion										
HCS	0.748									
HMO	0.544	0.757								
PTA	0.220	0.287	0.757							
PCM	0.558	0.454	0.441	0.778						
PPP	0.051	− 0.057	− 0.262	− 0.255	0.865					
PUS	0.429	0.392	0.586	0.592	− 0.273	0.795				
PCN	0.341	0.310	0.625	0.517	− 0.187	0.577	0.795			
UIA	0.413	0.390	0.504	0.639	− 0.135	0.554	0.565	0.747		
PPV	0.502	0.346	0.575	0.630	− 0.161	0.654	0.671	0.648	0.784	
ADA	0.208	0.129	0.171	0.198	0.142	0.282	0.221	0.274	0.295	1.000
Heterotrait-monotrait ratio (HTMT)—Matrix										
HCS										
HMO	0.712									
PTA	0.304	0.359								
PCM	0.706	0.573	0.546							
PPP	0.294	0.135	0.370	0.285						
PUS	0.535	0.501	0.743	0.746	0.325					
PCN	0.429	0.388	0.801	0.647	0.216	0.705				
UIA	0.551	0.517	0.653	0.841	0.182	0.716	0.711			
PPV	0.646	0.435	0.763	0.804	0.298	0.825	0.839	0.842		
ADA	0.253	0.144	0.203	0.224	0.157	0.312	0.247	0.313	0.329	

HCS: Health consciousness; HMO: Health Motivation; PTA: Perceived Technology Accuracy; PCM: Perceived critical mass; PPP: Perceived privacy protection; PUS: Perceived usefulness; PCN: Perceived Convenience; UIA: Use Intention of m-Health App; PPV: Perceived product value; ADA: Adoption of m-Health App.

Significant values are in italics.

### Hypothesis testing

Table 5 presents the results of the assessment of structural model. The results demonstrated insignificant relationship between HSC and UIA (*β*-value = 0.007; *t*-value = 0.140; *p*-value = 0.444). Similarly, the relationship between HMO and UIA (*β*-value = 0.065; *t*-value = 1.453; *p*-value = 0.073) were found not statistically significant. On the other hand, the following relationships were found statistically significant and positive: (1) the relationship between PTA and UIA (*β*-value = 0.134; *t*-value = 3.171; *p*-value = 0.001); (2) the relationship between PCM and UIA (*β*-value = 0.389; *t*-value = 8.235; *p*-value = 0.000); (3) the relationship between PPP and UIA (*β*-value = 0.073; *t*-value = 4.137; *p*-value = 0.000); (4) the relationship between PUS and UIA (*β*-value = 0.121; *t*-value = 2.055; *p*-value = 0.020); (5) the relationship between PCN and UIA (*β*-value = 0.201; *t*-value = 3.974; *p*-value = 0.001); (6) the relationship between UIA and ADA (*β*-value = 0.172; *t*-value = 5.084; *p*-value = 0.000); (7) the relationship between PPV and ADA (*β*-value = 0.216; *t*-value = 5.612; *p*-value = 0.001). Besides that, this study examined *f* ^2^ using the Cohen’s (1995) criterion: (1) *f* ^2^ of 0.35 denotes large effect size; (2) *f* ^2^ of 0.15 denotes medium effect size; (3) *f* ^2^ of 0.02 denotes small effect size.

**Table 5:** Path coefficients.

Hypo		Beta	*SE*	*t*	*CI (Min.)*	*CI (Max.)*	*p*	*r*^2^	*f*^*2*^	Decision
Factors intention to use e-doctor										
H1	HCS → UIA	0.007	0.052	0.14	− 0.082	0.090	0.444		0.006	Rejected
H2	HMO → UIA	0.065	0.045	1.453	− 0.006	0.143	0.073	0.515	0	Rejected
H3	PTA → UIA	0.134	0.042	3.171	0.068	0.208	0.001		0.019	Accepted
H4	PCM → UIA	0.389	0.047	8.235	0.305	0.460	0.000		0.148	Accepted
H5	PPP → UIA	0.073	0.018	4.137	0.042	0.100	0.000		0.009	Accepted
H6	PUS → UIA	0.121	0.059	2.055	0.035	0.228	0.020		0.014	Accepted
H7	PCN → UIA	0.201	0.051	3.974	0.111	0.277	0.001		0.043	Accepted
Factor affecting adoption to use e-doctor										
H8	UIA → ADA	0.172	0.034	5.084	0.119	0.229	0.000		0.016	Accepted
H9	PPV → ADA	0.216	0.039	5.612	0.151	0.277	0.000	0.101	0.029	Accepted
Mediating effect										
H10	HCS → UIA → ADA	0.001	0.009	0.136	− 0.014	0.016	0.446			Rejected
	HMO → UIA → ADA	0.011	0.008	1.396	− 0.001	0.025	0.081			Rejected
	PTA → UIA → ADA	0.023	0.008	2.859	0.011	0.038	0.002			Accepted
	PCM → UIA → ADA	0.067	0.015	4.374	0.043	0.094	0.000			Accepted
	PPP → UIA → ADA	0.013	0.004	2.985	0.006	0.020	0.001			Accepted
	PUS → UIA → ADA	0.021	0.012	1.677	0.005	0.046	0.047			Accepted
	PCN → UIA → ADA	0.035	0.011	3.232	0.017	0.052	0.001			Accepted
Moderating effect										
H11	PPV x UIA → ADA	0.026	0.018	1.472	0.000	0.058	0.007		0.003	Accepted

HCS: Health consciousness; HMO: Health Motivation; PTA: Perceived Technology Accuracy; PCM: Perceived critical mass; PPP: Perceived privacy protection; PUS: Perceived usefulness; PCN: Perceived Convenience; UIA: Intention to use m-Health App; PPV: Perceived product value; ADA: Adoption of m-Health App. SE: Standard Error, t: T statistics, *p*: probability/*p* value, Beta: path coefficient, *r*^*2*^ = R squared/determinant coefficient, *f*^*2*^ = effect size.

### Mediation and moderation effects

To assess the hypothesis of structural model, the *p*-value for the endogenous constructs, as well as the significance, and the sign of the path coefficients must be checked. Referring to Table 4, the results revealed that UIA did not mediate the relationship between ADA (β = 0.001, *p*-value = 0.446) and the relationship between HMO and ADA (β = 0.011, *p*-value = 0.081). Thus, indicating a rejection decision of *p*-value for behavioural to adopt m-health in this study. The results rejected the insignificant mediating effects of intention the link between HCS and HMO on ADA, as the mediating effects of intention the link between HCS and HMO on ADA as the *p*-value of HMO and HCS are greater than 0.05. However, UIA was found to mediate the following relationships: (1) the relationship between PTA and ADA (β = 0.023, *p*-value = 0.002); (2) the relationship between PCM and ADA (β = 0.067, *p*-value = 0.000).; (3) the relationship between PPP and ADA (β = 0.013, *p*-value = 0.001); (4) the relationship between PUS and ADA (β = 0.021, *p*-value = 0.047); (5) the relationship between PCN and ADA (β = 0.035, *p*-value = 0.001). Hence, these results indicating an acceptable decision of *p*-value for behavioural to adopt m-health in these findings. The results support the positive and significant effect mediating effects of intention the link between PUS, PCN, PCM, PPP, PUS, PCN on ADA, as the *p*-value of intention the link between PCM, PPP, PUS, PCN on ADA value is lower than > 0.05. Meanwhile, the moderating effect of PPV on the relationship between UIA and ADA (*β*-value = 0.026; *p*-value = 0.007) was confirmed in this study as *p*-value is lower than 0.05 (H11 accepted).

**Figure 3:**
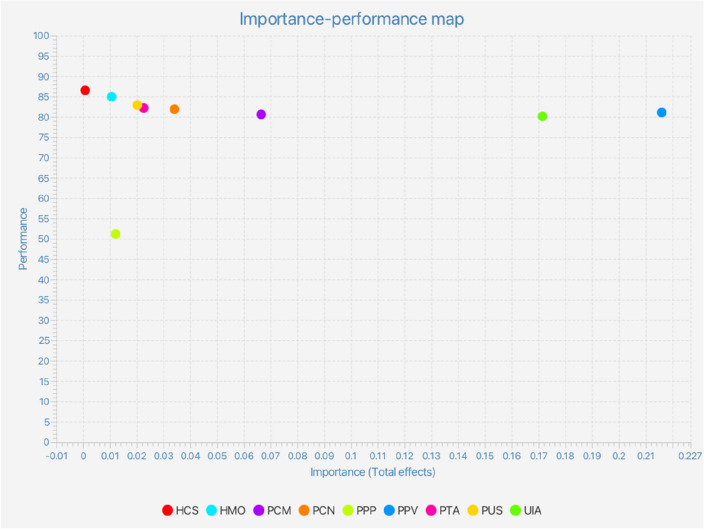
Importance-performance map.

Figure 3 presents the importance-performance map which graphically depicts the effects of the studied constructs. The “Performance/Index” under the “Final Results” presented the rescaled performance score of latent and manifest constructs, which demonstrated the disproportionate amount of weight of HCS, HMO, PCM, PCN, PPP, PPV, PTA, PUS, and UIA. As seen in Fig. 3, PPV and UIA exhibited the most substantial and important influence on ADA.

## Discussion

Focusing on identifying significant factors that influence consumers’ actual usage of m-Health app, this study quantitatively assessed the moderating role of perceived product value and mediating role of intention to use m-Health app among consumers in Indonesia. Firstly, this study empirically demonstrated the insignificant relationship between health consciousness and intention to use m-Health app (*p*-value = 0.444 > 0.05) and adoption to use m-Health app (*p*-value = 0.446 > 0.05). However, Iqbal et al.^59^ and Mustafa et al.^32^ reported otherwise. The prior study further highlighted the potential influence of consumers’ view of digital environment on their intention to use m-Health app, which was not explored in the current study.

Secondly, the current study found another insignificant relationship that involved health motivation and the intention to use m-Health app (*p*-value = 0.073 > 0.05) and HMO and adoption to use m-Health app (*p*-value = 0.081 > 0.05). Likewise, another prior study, specifically the study by Ezati^64^, reported otherwise; the study elaborated that consumers who consider that they are highly susceptible to illness are more likely to consider m-Health app as an important aspect to obtain the needed doctor consultation.

The current study’s results on the insignificant influence of health consciousness and health motivation on the intention to use m-Health app may reflect the low levels of health consciousness and health motivation among Indonesians. Besides that, this may be attributed to the dominance of respondents of under 25 years of age (> 50.0%) with lower education level (high school graduates; 33.7%)—they may exhibit lower levels of awareness and understanding towards healthcare. Meanwhile, the relationship between perceived technology accuracy and the intention to use m-Health app was found statistically significant (*p*-value = 0.001 < 0.005) and adoption to use m-Health app (*p*-value = 0.002 < 0.05)., which supported the results reported by Alam et al.^25^ and Ullah et al.^49^. This relationship may be explained by the intention is motivated by a desire of users to increase the use of m-Health and provides critical information for making crucial to their healthcare.

Another significant relationship was found between perceived critical mass and intention to use m-Health app (*p*-value = 0.000 < 0.05) and adoption to use m-Health app (*p*-value = 0.000 < 0.05). in the current study, which supported the results reported by Widyanto et al.^18^. The prior study elaborated that, when consumers believe that the majority of their peers are using a specific technology, critical mass may be reached following the increase in the propensity of actual usage of the technology. The current study also demonstrated the significant relationship between perceived privacy protection and the intention to use m-Health app (*p*-value = 0.000) and adoption to use m-Health app (*p*-value = 0.001 < 0.05). In regards to this, the notable observation of modern Internet users experiencing anxiety in their usage of the technology, and that such anxiety may exhibit substantial influence on their online privacy concerns^70^. The current study postulated the influence of (higher) education level and occupation (e.g., professionals) on consumers’ (strong) desire to address more online privacy concerns.

Adding to that, the relationship between perceived usefulness and the intention to use m-Health app (*p*-value = 0.020 < 0.005) and adoption to use m-Health app (*p*-value = 0.047 > 0.05)., which supported the results reported by Rawashdeh et al.^94^. The prior study highlighted the influence of perceived usefulness of m-Health app on the patients’ decision to use m-Health app. The current study found another significant relationship that involved perceived convenience and the intention to use m-Health app (*p*-value = 0.001 < 0.05) and adoption to use m-Health app (*p*-value = 0.001 < 0.05). The sample of this study was dominated by young consumers with high mobility in the metropolitan areas. All items of perceived convenience in this study, such as ease of use, time-savings, and flexibility, drove these respondents to consider using m-Health app for their convenience. Pal et al.^75^ reported similar findings and highlighted that perceived convenience and perceived danger, as significant factors that influence users’ decision-making, offset each other’s influence.

Most importantly, the current study empirically demonstrated the significant mediating role of the intention to use m-Health app in one’s decision of using m-Health app (*p*-value < 0.05), except for the cases of health consciousness and health motivation. The findings are consistent with the literature of UTAUT, that perceived technology Accuracy, perceived critical mass, perceived privacy protection, perceived usefulness, perceived convenience had important constructs influence intention and actual behaviour among users of m-health in Indonesia. The obtained results of the current study supported the study of Qing and Haiying^77^ on the mediating effect of behavioural intention on the influence of relevant constructs on the actual usage of m-Health app. However, this study has been unable to demonstrate that health consciousness and health Motivation in m-Health app to respondents in Indonesia. This result may be explained by the fact that Indonesian users are have low awareness to searching health information, learning, and using information channels. In other words, a possible explanation for the statistic results may be the lack of education and motivation regarding health consciousness to adopt m-health as digital platform to prevent against chronic diseases earlier as well as consult with a doctor remotely and cheaper.

The current study also empirically demonstrated the significant moderating effect of perceived product value on the relationship between the intention to use m-Health app and actual usage of m-Health app (*p*-value = 0.007 < 0.05). These results supported the study of Shah et al. ^52^ on how perceived product value can enhance consumers’ happiness, customer loyalty, customer retention, and ultimately, business success. The actual value of the price of a product indeed affects consumers’ purchasing decision, which may be indicative of their desire for the product. When both customer intention and customer perceived value are high, the adoption of m-Health will have high by customers. In order to improve m-Health app, however, the website need to spend much on maintenance and renewal, which may cost too much in order to earn a profit. From the result of moderating effect of customer perceived value, this study discovers that when m-Health app offers acceptable the quality of the application and emphasize improving customer perceived value, they will gain higher customer intention to use m-Health. According to this finding, this study assumes that customer perceived value is a key factor to affect customer intention.

## Theoretical implication

The emergence of mHealth-related applications has brought a complex breakthrough in the traditional healthcare industry, and many scholars have explored consumer and user perceptions and acceptance of mHealth digital technology in lots of countries (such as China, Malaysia and Saudi Arabia) through TAM and the extended model-UTAUT^5,27,95,96^. Thus, the above studies have confirmed the feasibility of the UTAUT model in exploring healthcare digital technology adoption behaviors and user acceptance. Accordingly, this study successfully extends the UTAUT framework in the context of digital behavior and mHealth digital technology adoption and considers and incorporates several important factors such as perceived privacy protection and perceived compatibility. Based on the findings of this study, UTAUT plays an important role in elucidating technology and social behaviours within the Asian context. The inclusion of other potential determinants can expand the existing theory, such as gender and education level. The current study also presented empirical evidence on the sustainable use of m-Health app, specifically the mediating role of intention to use m-Health app and the moderating role of perceived product value. Unlike other prior studies, the current study obtained evidence on the full moderating effect of perceived product value. This study also established the relationship between consumers’ expectations and perceptions, which expanded the overall theoretical framework on the adoption of m-Health app.

## Practical implications

From the practical perspective, the current study demonstrated the potential influence of m-Health app on the consumer behaviour in Indonesia. Also considering the needs and perceptions of consumers and users of m-health apps, m-Health apps should provide two-way communication, engage consumers, and provide necessary knowledge support. Hermann et al.^97^ found that m-Health apps enabled by digital technologies can be categorized according to the patient needs they address in healthcare: diagnosis, prevention, treatment, adherence, lifestyle, and patient engagement. Multiple groups, including consumers, patients, providers of medical services, software developers, governments, and even non-governmental organizations, are united by mobile technology. Consequently, mobile communication innovations should strive to enhance access to health care and its delivery, patient engagement, and clinical outcomes. The widespread availability of mobile Web access permits developers to increase their customer-base. However, the continuous and expanding supply of m-Health applications raises market expectations. M-health developers must contend with a limitation associated with human perception in general. Once medical information is presented, there is a possibility that nearly half of those who read it will be unable to comprehend it. Throughout the duration of the designing process, designers should strive for maximum readability and efficiency. To achieve this, they must consider the quantity of information that the patient, the consumer, and the clinician must peruse and access. Accordingly, the development and improvement of the features of m-Health app can consider high accessibility, ease of use, ease of monitoring, reliable support system, ease of acquiring information, and information update. Furthermore, the government should consider of learning initiatives for consumers to gain knowledge on the use of mobile applications and provide the necessary support for the expansion of digital applications for the benefits of consumers. Both social media and government sector advocacy can strengthen the awareness and acceptance of mHealth apps among consumers and the user population and create a desire to try and use them after learning information about their privacy protection and wide range of uses, which in turn can lead to a willingness to manage their own health and access medical assistance through mHealth apps.

## Conclusion

Overall, the current study presented novel insights on the significant influence of perceived product value and intention to use m-Health app among consumers. Furthermore, this study empirically proved the significant and substantial influence of perceived critical mass, perceived convenience, perceived technology accuracy, and perceived privacy protection on the actual usage of m-Health app via the intention to use m-Health app as a mediator. This study also empirically demonstrated the moderating role of perceived product value in enhancing the relationship between intention to use m-Health app and actual usage of m-Health app. The outcomes of the importance-performance map in this study identified perceived product value and intention to use m-Health as the most significant determinants of the actual usage of the technology. The adequacy of UTAUT in elucidating the intention to use m-Health app and actual usage of m-Health app among consumers in Indonesia was confirmed in this study. Conclusively, this study presented significant theoretical and practical implications on the adoption of m-Health app and the significance of intention to use m-Health app and the actual usage of m-Health app.

## Limitation

The current study encountered several limitations. Firstly, the use of cross-sectional research design in the current study resulted in limited generalisability of findings. Therefore, it is recommended for future research to make use of longitudinal data for more comprehensive findings. Secondly, the current study had limited data on digital behaviour, which can be explored in future research, particularly on the use of mobile payment services. Thirdly, the implementation of convenience sampling strategy may have limited the distribution of sample, particularly middle-income consumers. Therefore, it is recommended for future research to expand on this aspect. Lastly, considering the risk-propensity nature and sensitivity to monetary losses, it is recommended for future research to replicate the current study to examine the influence of risk on emerging economies that may manifest itself in various ways.

### Supplementary Information

Supplementary Information 1.

Supplementary Information 2.

## Data Availability

The original contributions presented in the study are included in the article/Supplementary Material, further inquiries can be directed to the corresponding author/s.
