# Supplementary material for: Predicting m-health acceptance from the perspective of unified theory of acceptance and use of technology
Source: Sci Rep. 2024 Jan 3;14:339. doi: 10.1038/s41598-023-50436-2 (PMC10764358; doi:10.1038/s41598-023-50436-2)
Supplement: Supplementary file 2 — Supplementary Information 2. [file 41598_2023_50436_MOESM2_ESM.docx]

**Supplementary Material.** Survey Instrument

| Code | Items |
| --- | --- |
| HSC1 | I think my health depends on how well I take care of myself. |
| HSC3 | I think taking preventive measures help to stay healthy. |
| HSC4 | Living a healthy life is important to me. |
| HSC5 | I am constantly examining my health. |
| HMO1 | I usually value my health. |
| HMO2 | I have good knowledge to prevent health issues. |
| HMO3 | I try to prevent health problems before I feel any symptoms. |
| HMO5 | I am concerned about health hazards and try to take action to prevent them |
| PTA1 | I think I can rely on the health services provided by m-Health Apps. |
| PTA2 | I think the m-Health App delivers consistent results over time. |
| PTA3 | I think m-Health App have good working standards continuously. |
| PTA5 | I feel confident that m-Health Apps are offering error-free results. |
| PCM1 | Most people in my neighborhood are using the m-Health App. |
| PCM2 | Many people to whom I usually communicate are using the m-Health App. |
| PCM4 | I know many people having health issues are using the m-Health App regularly. |
| PCM5 | m-Health App devices are gaining popularity. |
| PPP1 | It would be risky to disclose my personal health information to vendors providing m-Health Apps. |
| PPP2 | There would be a high potential for loss associated with disclosing my personal health information to vendors providing m-Health Apps. |
| PPP3 | There would be too much uncertainty associated with giving my personal health information to vendors providing m-Health Apps. |
| PPP4 | Disclosing personal information to a third party is risky. |
| PUS1 | Using the m-Health App enables me to check my health condition quickly. |
| PUS2 | Using the m-Health App makes it easier to check my health condition. |
| PUS3 | Using the m-Health App save my time and effort. |
| PUS4 | m-Health App is beneficial to manage health. |
| PCN1 | It is easy to use m-Health Apps. |
| PCN1 | I find using m-Health Apps save my time. |
| PCN1 | Using the m-Health App is convenient. |
| PCN1 | I can use the m-Health App at any time. |
| UIA1 | I intend to use m-Health apps to manage my health in the future. |
| UIA2 | I will always try to use m-Health apps to manage my health in my daily life in the future. |
| UIA3 | I plan to use m-Health apps frequently to manage my health in the future. |
| UIA5 | I predict I will use m-Health apps to manage my health information |
| PPV1 | m-Health Apps offer good value for money. |
| PPV2 | Using m-Health Apps are beneficial |
| PPV4 | I think the m-Health App is worthwhile. |
| PPV5 | Overall, using the m-Health App delivers good value to me. |
| ADA1 | How often do you use m-Health App? |

**Notes:** HCS: Health consciousness; HMO: Health Motivation; PTA: Perceived Technology Accuracy; PCM: Perceived critical mass; PPP: Perceived privacy protection; PUS: Perceived usefulness; PCN: Perceived Convenience; UIA: Use Intention of m-Health App; PPV: Perceived product value; ADA: Adoption of m-Health App.
